# Infection of Epstein–Barr Virus in Type III Latency Modulates Biogenesis of Exosomes and the Expression Profile of Exosomal miRNAs in the Burkitt Lymphoma Mutu Cell Lines

**DOI:** 10.3390/cancers10070237

**Published:** 2018-07-19

**Authors:** Asuka Nanbo, Harutaka Katano, Michiyo Kataoka, Shiho Hoshina, Tsuyoshi Sekizuka, Makoto Kuroda, Yusuke Ohba

**Affiliations:** 1 Faculty of Medicine and Graduate School of Medicine, Hokkaido University, N15 W7, Kita-ku, Sapporo 060-8638, Japan; yohba@med.hokudai.ac.jp; 2 Department of Pathology, National Institute of Infectious Diseases, 1-23-1 Toyama, Shinjuku-ku, Tokyo 162-8640, Japan; katano@nih.go.jp (H.K.); michiyo@nih.go.jp (M.K.); shihozaru2002@yahoo.co.jp (S.H.); 3 Department of Pediatrics, Yachiyo Medical Center, Tokyo Women’s Medical University, 477-96, Owada-Shinden, Yachiyo, Chiba 276-0046, Japan; 4 Pathogen Genomic Center, National Institute of Infectious Diseases, 1-23-1 Toyama, Shinjuku-ku, Tokyo 162-8640, Japan; sekizuka@nih.go.jp (T.S.); makokuro@niid.go.jp (M.K.)

**Keywords:** Epstein–Barr virus, Burkitt’s lymphoma, exosomes, miRNA, next-generation sequencing

## Abstract

Infection of Epstein–Barr virus (EBV), a ubiquitous human gamma herpesvirus, is associated with various malignancies in B lymphocytes and epithelial cells. EBV encodes 49 microRNAs in two separated regions, termed the BART and BHRF1 loci. Although accumulating evidence demonstrates that EBV infection regulates the profile of microRNAs in the cells, little is known about the microRNAs in exosomes released from infected cells. Here, we characterized the expression profile of intracellular and exosomal microRNAs in EBV-negative, and two related EBV-infected Burkitt lymphoma cell lines having type I and type III latency by next-generation sequencing. We found that the biogenesis of exosomes is upregulated in type III latently infected cells compared with EBV-negative and type I latently infected cells. We also observed that viral and several specific host microRNAs were predominantly incorporated in the exosomes released from the cells in type III latency. We confirmed that multiple viral microRNAs were transferred to the epithelial cells cocultured with EBV-infected B cells. Our findings indicate that EBV infection, in particular in type III latency, modulates the biogenesis of exosomes and the profile of exosomal microRNAs, potentially contributing to phenotypic changes in cells receiving these exosomes.

## 1. Introduction

Epstein–Barr virus (EBV), a human gamma herpesvirus, contributes causally to lymphomas and epithelial malignancies, such as Burkitt lymphoma (BL), Hodgkin lymphoma (HL), nasopharyngeal carcinoma (NPC), gastric carcinoma (GC), and post-transplant lymphoproliferative disorders (PTLD) [1]. The expression pattern of EBV-encoded latent genes defines three latency types specific to individual EBV-associated tumors [1]. Latency type I is associated with BL and GC and is restricted to the expression of EBV-encoded nuclear antigen 1 (EBNA1), the EBV-encoded small RNAs (EBERs), and BamHI A rightward transcripts (BARTs). Latency type II, which is associated with HD and NPC, expresses EBNA1, both EBERs, BARTs, and the latent membrane proteins (LMP1, LMP2A and LMP2B). Latency type III, which is characteristic of PTLD and EBV-transformed lymphoblastoid cell lines (LCLs), expresses both the transcripts and all the EBV latent proteins, including the six nuclear antigens (EBNA1, EBNA2, EBNA3A, EBNA3B, EBNA3C and EBNA-LP), and three membrane proteins (LMP1, LMP2A and LMP2B).

EBV-infected cells have been shown to secrete exosomes [2,3]. Exosomes are extracellular micro vesicles (50–100 nm in diameter) that originate from the luminal membranes of multivesicular bodies (MVBs), which bud off parts of their membrane into their lumen to form intraluminal vesicles and are extracellularly released when MVBs fuse with the plasma membrane [4,5]. Exosomes are secreted from various cell types, into body fluids, including blood, urine, saliva, breast milk, cerebrospinal fluid, and ascites [6,7,8]. Exosomes possess various functions in adaptive immune responses and also the development of malignancies by transferring sets of proteins, lipids and RNAs to the recipient cells [6,7,9]. It has been shown that exosomes carry a distinctive repertoire of microRNAs (miRNAs), other small non-coding RNAs, natural antisense RNAs, tRNAs, mRNAs and long non-coding RNAs [10,11,12,13,14]. 

miRNAs are short 15–25 nucleotides in length, non-coding RNAs, which mediate post-transcriptional gene silencing by binding to the 3′-untranslated region (UTR) or open reading frame (ORF) region of target mRNAs. The involvement of miRNAs in many biological activities has been well documented, including cell proliferation, cell differentiation, cell migration, disease initiation, and disease progression [15,16]. EBV encodes 25 pre-miRNAs of which 3 are in the BHRF locus and 22 in the BART locus [17]. Accumulating evidence demonstrates that mature EBV miRNAs contribute to the establishment of EBV infection and induction of EBV-associated tumors by modifying host and viral functions in B cells [18,19,20,21,22,23] and epithelial cells [24,25,26,27,28]. It has been shown that EBV infection also modulates the expression of specific set of cellular miRNAs [29,30,31], such as miR-19, miR-21, miR-23a, miR-24, miR-146a and b, and miR-155, which are known to associate with tumor progression [32]. 

EBV-encoded miRNAs are transferred to target cells through exosomes [33,34] and subsequently regulate their targets [33]. A recent study has demonstrated that oropharyngeal epithelial cells release exosomes containing the epithelium-specific miRNA, miR-200, which are subsequently transferred to cocultured latently EBV-infected BL cells and induce viral reactivation [35].

Not all miRNAs found in a cell are incorporated into exosomes [36]. Moreover, the expression of host and viral miRNAs are also affected in the different types of EBV latency [29,30,31]. Therefore, it is important to characterize the expression profile of cell and viral miRNAs incorporated in the exosomes to elucidate the role of exosomes derived from EBV-infected cells in the viral lifecycle. Some studies have analyzed the expression profiles of host and/or EBV miRNAs in EBV-associated tumor tissues [24,31,37,38,39,40,41,42,43] and cell lines [29,44] by microarray and real-time quantitative PCR (qPCR). Because of the limitation of these technologies, both miRNAs or other RNA species were often undetected. In contrast, next-generation sequencing (NGS) is a powerful tool for characterization of the expression profile of small RNAs. This approach has revealed the expression profiles of host and viral miRNAs in EBV-associated malignancies [45,46,47,48,49]. However, little is known about expression profiles of host and viral miRNAs in exosomes released from EBV-infected cells [30,50]. 

Here, we isolated exosomes derived from three BL cell lines possessing closely related genetic backgrounds. Mutu I and Mutu III cell are type I and type III latently infected cells, respectively established from the same African BL tumor. The EBV-negative subclone (Mutu^−^) was isolated from Mutu I by the limiting dilution methods. We characterized the expression profile of intracellular and exosomal miRNAs derived from individual Mutu cells by NGS. We observed that the biogenesis of exosomes is upregulated in type III latently infected cells having a low number of EBV DNA molecules. We also found that more EBV miRNAs are in Mutu III cells and exosome (III) than in EBV-negative and type I infected cells and their exosomes. Moreover, multiple specific miRNAs were predominantly incorporated into exosomes derived from Mutu III cells. Finally, stem-loop real-time RT-PCR revealed that several viral miRNAs were transferred to the EBV-negative GC cells via exosomes. Our findings indicate that EBV infection, in particular in type III latency, modulates both biogenesis of exosomes and the expression profile of exosomal miRNAs, which may contribute to phenotypic changes in recipient cells.

## 2. Results

### 2.1. Infection with EBV Is Sufficient to Accelerate the Biogenesis of Exosomes in Mutu III Cells

**Figure 1:**
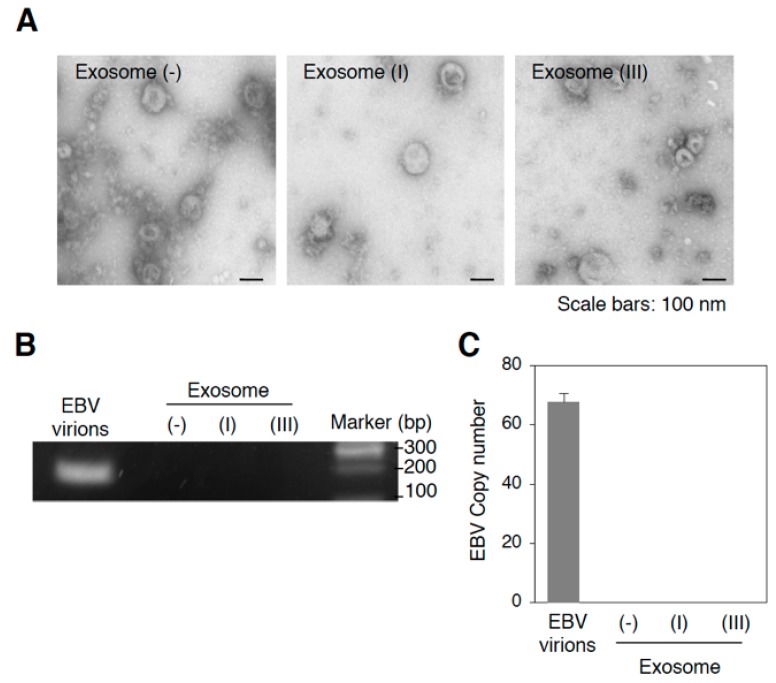
Isolation of exosomes released from Mutu cells. (**A**) Representative electron micrographs of isolated exosomes from Mutu cells. Isolated Exosome (−), exosome (I), or exosome (III) by ultracentrifugation were analyzed by electron microscopy. Scale bars: 100 nm; (**B**) Detection of EBV DNA in the isolated exosomes by PCR. DNA was isolated from DNase-treated culture medium containing EBV virions and isolated exosomes followed by PCR. EBV-encoded *BALF5* gene was amplified. PCR products were subjected to agarose gel electrophoresis; (**C**) Detection of EBV DNA in the isolated exosomes by real-time PCR. DNA was isolated from DNase-treated culture medium containing EBV virions and isolated exosomes followed by real-time PCR. EBV-encoded *BALF5* gene was amplified. The experiment was performed three times independently and the average and its SD are shown in each condition.

**Figure 2:**
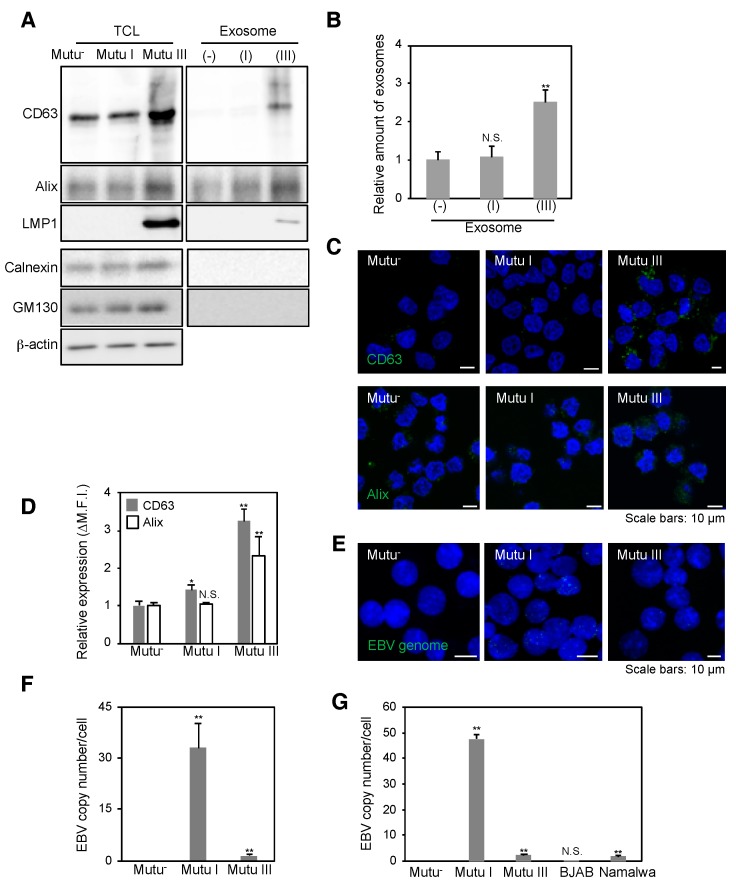
Infection with limited EBV copies is sufficient to promote the biogenesis of exosomes in Mutu III cells. (**A**) Western blot analysis of isolated exosomes. Total cell lysates (TCL; left) and isolated exosomes (right) obtained from Mutu cells were subjected to western blot with antibodies against CD63, Alix, LMP1, Calnexin, GM130, and β-actin; (**B**) Analysis of amounts of protein in isolated exosomes released from Mutu cells. Isolated exosomes were subjected to a Bradford protein assay. Relative amounts of protein are shown. The experiment was performed three times independently and the average and its SD are shown in each condition. N.S., not significant. ** *p* < 0.01 vs. respective control (Student’s *t*-test); (**C**) Expression of CD63 and Alix in Mutu cells. The distribution of CD63 (top, green) or Alix (bottom, green) in Mutu^−^ (left), Mutu I (middle) and Mutu III cells (right) was analyzed by immunofluorescence staining. The nuclei (blue) were counterstained with Hoechst 33342. Scale bars: 10 µm; (**D**) Flowcytometric analysis of expression of CD63 and Alix in Mutu cells. Expression of CD63 or Alix in Mutu cells was measured by flowcytometry. The experiment was performed three times independently and the average and its SD are shown in each condition. N.S., not significant. * *p* < 0.05, ** *p* < 0.01 vs. respective control (Student’s *t*-test); (**E**) FISH analysis of EBV genome in Mutu cells. Mutu^−^ (left), Mutu I (middle) and Mutu III cells (right) were subjected to FISH with a specific probe targets EBV genome. Individual EBV genomes are shown in green. The nuclei (blue) were counterstained with Hoechst 33342. Scale bars: 10 µm; (**F**) Measurement of EBV copy numbers in Mutu cells. Four fields containing 10–20 nuclei were selected randomly and numbers of EBV genomes per cell were analyzed. Average and its SD are shown in each condition. ** *p* < 0.01 vs. respective control (Student’s *t*-test); (**G**) A real-time PCR analysis of EBV copy numbers in Mutu cells. DNAs were isolated from Mutu^−^, Mutu I, Mutu III, BJAB, or Namalwa cells. EBV titers were analyzed by quantitative PCR. EBV-encoded *BALF5* gene was amplified. As an internal control, the human rhodopsin gene was used. The experiment was performed three times independently and the average and its SD are shown in each condition. ** *p* <0.01 vs. respective control (Student’s *t*-test).

To perform NGS analysis for exosomal miRNAs, we purified exosomes released from EBV-negative (Mutu^−^), EBV-infected of type I (Mutu I), or type III latency (Mutu III) [exosome (−), exosome (I), or exosome (III), respectively] by ultracentrifugation through a sucrose cushion. Because some BL cell lines support the viral lytic cycle [51], we tested for EBV virions in the isolated exosomal fractions by electron microscopy (Figure 1A) and real-time PCR for EBV-encoded DNA polymerase BARF5 gene (Figure 1B,C) and confirmed their absence. We also measured the presence of exosome markers, such as CD63 [52] and ALG-2-interacting protein X (Alix) [53] in Mutu cells and exosomal fractions by western blot analysis. We observed the highest expression of CD63 and Alix in Mutu III and exosome (III) (Figure 2A). In contrast, exosome fractions were negative for calnexin (an endoplasmic reticulum marker) and GM130 (a Golgi marker), which are commonly used as negative controls of extracellular vesicles [54]. 

We also confirmed that LMP1 is incorporated into exosome (III) (Figure 2A), which is consistent with previous observations by us and the other groups [55,56,57,58]. Moreover, a Bradford protein assay showed approximately twice as many exosomes (III) than exosome (−) and exosome (I) (Figure 2B). We also examined the formation of MVBs, where the biogenesis of exosomes take place, in individual Mutu cell lines by immunofluorescence staining. Mutu III cells formed MVBs more than Mutu^−^ and Mutu I cells (Figure 2C(top),D), indicating that EBV infection in type III latency promotes biogenesis of exosomes. We also analyzed the expression of Alix, which is associated with the endosomal sorting complex required for transport (ESCRT) and is involves in the biogenesis of exosomes. Alix was visualized as speckles in the cytoplasm of Mutu cells. Immunofluorescence staining revealed that Mutu III cells expressed Alix more than Mutu^−^ and Mutu I cells (Figure 2C(bottom),D). Finally, we analyzed the copy numbers of EBV genomes maintained in Mutu cells by FISH (Figure 2E,F) and real-time PCR (Figure 2G). As expected, no EBV genomes was detected in Mutu^−^ cells. Whereas approximately 30–45 copies of EBV genomes on average were maintained in individual Mutu I cell, approximately 2–3 copies of EBV genomes were observed in Mutu III cell (Figure 2E–G). These data indicate that infection with limited copy numbers of EBV is sufficient to accelerate the biogenesis of exosomes in Mutu III cells. 

### 2.2. More EBV miRNAs Are in Mutu III Cells and Exosomes (III) Than in EBV-Negative and Type I Latently Infected Cells and Their Exosomes

To determine the effect of EBV infection on miRNA expression profile, a small RNA-enriched fraction was isolated from cells and exosomes, respectively. The isolated RNAs were then subjected to NGS, which yielded 119,769, 26,388, and 1,559,462 reads for RNAs isolated from Mutu^−^, Mutu I and Mutu III, respectively. On the other hand, 66,018, 535,527 and 2,445,816 reads were obtained from the RNAs isolated from exosome (−), exosome (I), and exosome (III), respectively. The alignment of the sequences to the reference genome resulted in the annotation of 12.64–67.34% of cellular miRNA reads and 44.11–80.96% of exosomal miRNA reads (Table 1). Because exosomes are known to contain a substantial amount of tRNA and its fragments, the sequences were also aligned to the human-tRNA database. 2.1–4.65% of cellular RNAs and 2.33–3.97% of exosomal RNAs were characterized as human tRNAs (Table 1). The sequences were also aligned to miRBase. 7.14% and 3.85% of the annotated cellular and exosomal miRNAs, respectively, originated from EBV in Mutu I cells. In Mutu III, 14.44% of annotated miRNAs in the cells originated from EBV, whereas the 8.07% of annotated exosomal miRNAs were derived from EBV (Table 1). These results indicate that more EBV miRNAs are in Mutu III cells and exosomes (III) than in EBV-negative and type I latently infected cells and their exosomes.

### 2.3. Characterization of Expression Profile of Cellular and Exosomal miRNAs of Mutu Cells

**Figure 3:**
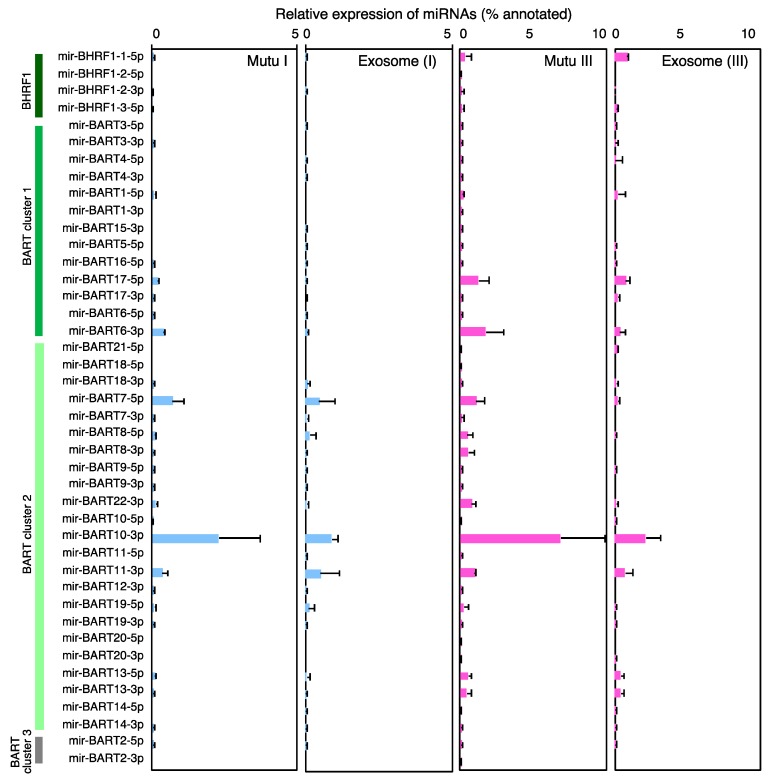
Expression profile of EBV-encoded miRNAs in Mutu cells and exosomes. RNAs isolated from Mutu I, Mutu III cells, exosome (I), and exosome (III) were subjected to NGS. Analysis was duplicated for intracellular miRNAs derived from Mutu cells and exosome (III), and triplicated for exosome (I). Average ratio of miRNA reads to annotated total miRNA reads and its SD are shown.

The annotated miRNAs from the human genome derived from Mutu cells and their exosomes were then ranked according to their read counts. Several host miRNAs such as miR-92a-1//miR-92a-2-3p, miR-148a-3p, miR-181a-2//miR-181a-1-5p, miR-182-5p, and miR-378a-3p were ubiquitously and abundantly observed both in the cells and their exosomes. Moreover, various oncogenic miRNAs involved in cell cycle progression (e.g., miR-21-3p, miR-25-3p, miR-30d-5p, miR-92a-1//miR-92a-2-3p, miR-181a-2//miR-181a-1-5p, and miR-181b-1//miR-181b-2-5p), and in the apoptotic pathway (e.g., miR-21-3p, miR-181a-2//miR-181a-1-5p, let-7a-1//let-7a-2//let-7a-3-5p, and miR-92a-1//miR-92a-2-3p) [32] were also expressed ubiquitously both in the cells and the exosomes (Table 2 and Table 3, Table S1). In contrast, miR-155, which has been shown to be associated with B cell lymphomagenesis, were detected at this level only in Mutu III cells and exosome (III). Multiple, specific host miRNAs including miR-143-3p, miR-877-5p, miR-4516-5p, miR-6087-5p, and miR-7704-5p were found in the top 40 miRNAs in exosome (III). Four (miR-BART6-3p, miR-BART7-5p, miR-BART10-3p, and miR-BART11-3p) and 3 (miR-BART7-5p, miR-BART10-3p, and miR-BART11-3p) EBV miRNAs ranked in the top 40 miRNAs in Mutu I cells and exosomes (I), respectively. On the other hand, 6 (miR-BART6-3p, miR-BART7-5p, miR-BART11-3p, miR-BART13-5p, miR-BART17-5p, and miR-BART22-3p) and 9 viral miRNAs (miR-BART6-3p, miR-BART7-5p, miR-BART10-3p, miR-BART11-3p, miR-BART13-5p, miR-BART17-5p, miR-BART19-5p, miR-BART22-3p, and miR-BHRF1-1-5p) ranked in the top 40 miRNAs in Mutu III cells and exosomes (III), respectively (Table 2 and Table 3, Table S1, Figure 3). Among EBV miRNAs, miR-BART10-3p was detected predominantly in Mutu I (3.74%), Mutu III (8.48%), and exosomes (III) (4.2%). miR- BHRF1-1-5p was specifically detected in exosome (III) (0.26%). All these data demonstrate that infection of EBV in type III latency leads to the specific expression profiles of host and viral miRNAs in the cells and in their exosomes.

### 2.4. The Role of EXOmotifs in Sorting of Specific miRNA into Exosomes

**Figure 4:**
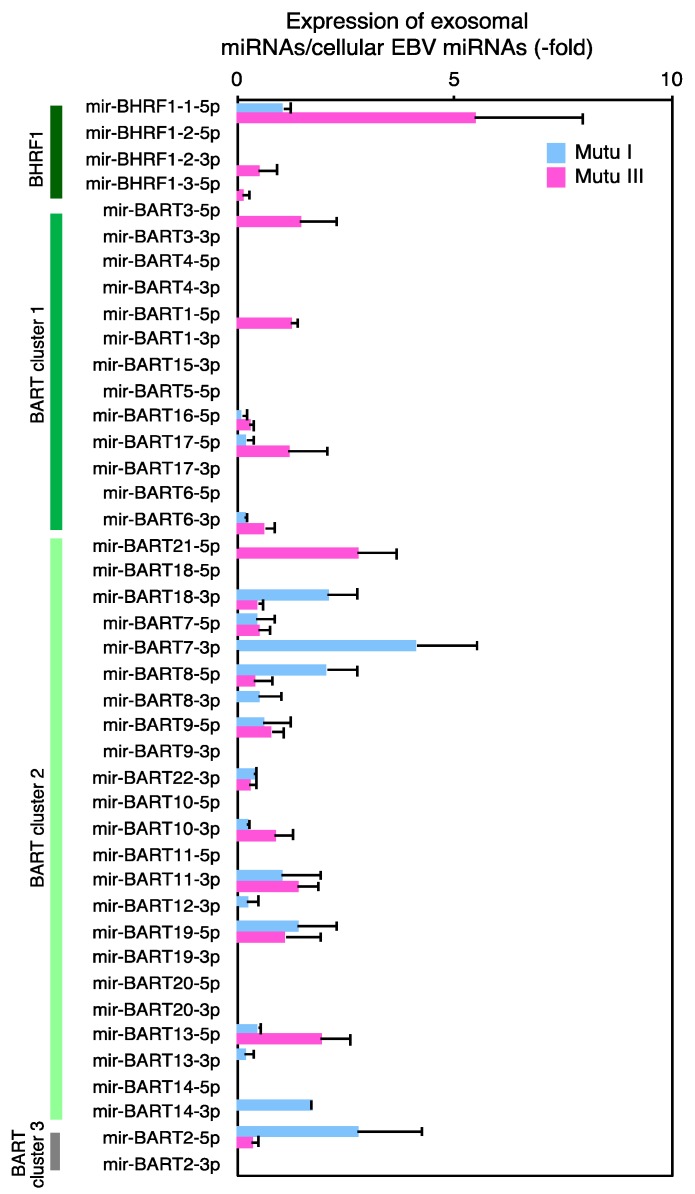
Expression ratio of exosomal to cellular EBV miRNAs. RNAs isolated from Mutu I, Mutu III cells, exosome (I), and exosome (III) were subjected to NGS. Ratio of exosomal to cellular EBV miRNA reads are shown. Results of Mutu I and Mutu III shown in blue and pink, respectively. Analysis was duplicated and average and its SD are shown.

NGS analysis revealed that multiple cellular miRNAs, such as miR-10b-5p, miR-486-1//miR-486-2-5p, miR-4516-5p, miR-6087-5p, and miR-7704-5p were significantly concentrated (more than 100-fold) in exosomes (III) (Table 3 and Table S2). Among viral miRNAs, miR-BART7-3p and BART2-5p, or miR-BHRF1-1-5p were highly concentrated in exosome (I) or exosomes (III), respectively (Figure 4, Table 3 and Table S3). To date, several mechanisms by which specific miRNAs are sorted to exosomes have been considered. It has been shown that sumoylated heterogeneous nuclear ribonucleoprotein A2B1 (hnRNPA2B1) recognizes consensus sequence motifs, EXOmotifs within miRNAs, and guides their sorting to exosomes [59]. Therefore, we analyzed the sequences of host miRNAs that were concentrated more than 100-fold in exosome (III). We also examined the sequences of viral miRNAs which were concentrated more than 5-fold in exosomes. We found that host miRNAs concentrated in exosomes possessed 1–8 EXOmotifs in their sequences (Table 4). In particular, miR-6087-5p, one of most concentrated miRNAs, possessed 8 EXOmotifs (four GGGG, two GGCG, one GGGC, and one TGAG motifs). We also identified two or one EXOmotifs in miR-BHRF-1-1-5p and miR-BART7-3p, or miR-BART2-3p, respectively (Table 4). Taken together, EXOmotifs likely, partly contributed to the efficient sorting of specific miRNAs into exosomes, which helps to explain the asymmetric distribution of cellular and exosomal miRNAs of Mutu cells.

### 2.5. Exosome-Mediated Transfer of EBV miRNAs to Target Epithelial Cells

**Figure 5:**
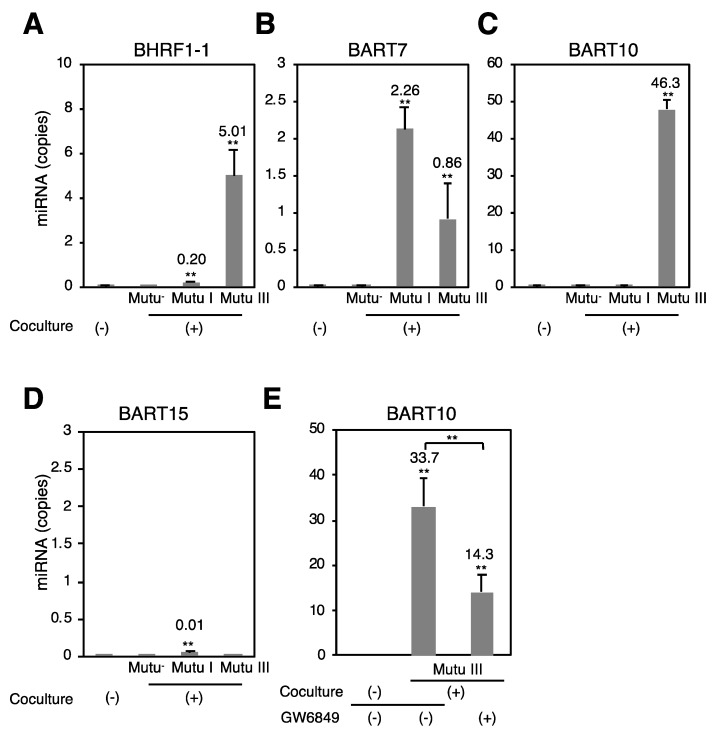
Transfer of EBV miRNAs to the recipient cells cocultured with Mutu cells. Mutu^−^, Mutu I, or Mutu III cells were cocultured with AGS cells through trans-well inserts for 72 h. RNAs were isolated from AGS cells and subjected to stem-loop RT-PCR to analyze copy numbers of miR-BHRF1-1 (**A**); miR-BART7 (**B**); miR-BART10 (**C**); and miR-BART15 (**D**); To determine the role of exosomes in transfer of miR-BART10 to recipient cells, Mutu III cells were cocultured with AGS cells in the presence or absence of GW4869 (**E**). The experiment was performed three times independently and the average and its SD are shown in each condition. The average copy number of miRNA is shown. ** *p* < 0.01 vs. respective control (Student’s *t*-test).

Finally, we examined whether EBV miRNAs expressed in Mutu cells are transferred to recipient cells via exosomes. We cocultured Mutu cells with EBV-negative human GC AGS cell lines separately through a trans-well for 3 days. Total RNA was isolated from cocultured AGS cells and subjected to stem-loop real-time RT-PCR to quantify the copy numbers of miR-BHRF1-1, miR-BART7, miR-BART10, and miR-BART15. As expected, no EBV miRNAs were detected in uncocultured AGS cells and AGS cells cocultured with Mutu^−^ cells (Figure 5). miR-BHRF1-1 and miR-BART10 were predominantly detected in AGS cells cocultured with Mutu III (Figure 5A,C). miR-BART7 was detected more efficiently in AGS cells cocultured with Mutu I cells relative to those cocultured with Mutu III (Figure 5B). Little miR-BART15 was observed in the cocultured recipient cells (Figure 5D). The frequency of the expression of miRNAs in the recipient cells was consistent with their expression levels in the exosomes, as determined by NGS (Figure 3). To confirm the importance of the exosome secretion from Mutu cells, the cocultured cells were treated with GW4869, an inhibitor of sphingomyelinase that markedly reduces exosome secretion [60]. GW4869 inhibited transfer of BART10 to AGS cells cocultured with Mutu III (Figure 5E), indicating that transfer of EBV miRNAs to recipient cells was mediated by exosomes. Taken together, these data indicate that type III latency of EBV infection modulates the expression profile of host and viral miRNAs in cells and exosomes, which may contribute to induction of EBV-associated malignancies or other pathologies in the recipient cells.

## 3. Discussion

Here, we have characterized the expression profile of cellular and exosomal miRNAs derived from cell lines originating from the same African BL patient with different states of EBV-infection by next-generation sequencing. 

Both the formation of MVBs and the biogenesis of exosomes were upregulated in Mutu III cells, which have only a low number of EBV genomes (Figure 2). Hurwitz and colleagues demonstrated that CD63 plays a critical role in LMP1-mediated enhancement of exosome production [58]. The same group recently observed that CD63 coordinates the autophagic and endosomal pathways to regulate LMP1-mediated signals and secretion of exosomes [61]. Previously we demonstrated that EBV-infected cells require a certain threshold number of EBV genomes for their optimal growth under selection [62], suggesting that maintenance of limited copy numbers of EBV is sufficient to accelerate LMP1-mediated exosome production. 

Exosome (III) contain more viral miRNAs than exosome (−) and exosome (I). Moreover, multiple specific cellular miRNAs were predominantly incorporated into exosomes (III) (Table 3). Although EXOmotifs were frequently identified in the highly concentrated miRNAs in exosome (III) (Table 4), the numbers of EXOmotifs varied among these miRNAs and no significant correlation was found between sorting efficiency of miRNAs to the exosomes and their number of EXOmotifs, suggesting that EXOmotifs-independent mechanism(s) for sorting of miRNA to exosomes are likely involved. For instance, Kosaka et al. demonstrated that the neural sphingomyelinase 2 (nSMase2) upregulates the efficiency of sorting of miRNAs to the exosomes [63]. Other studies suggest a possible mechanism involving miRNA sorting in a miRNA 3′ end nucleotide or miRNA induced silencing complex (miRISC)-dependent manner [37]. 

Multiple specific cellular miRNAs, such as miR-143, miR-877, miR-4516-5p, miR-6087-5p, and miR-7704-5p were incorporated into exosome (III) (Table 3). miR-143 has been characterized as a tumor-suppressive factor by targeting several oncogenes, including Kirsten rat sarcoma viral oncogene homolog (KRAS) and extracellular signal-regulated kinases 5 (ERK5) [64]. Two independent reports demonstrate a role for miR-877 as a tumor suppressor in renal cell carcinoma by targeting eukaryotic elongation factor-2 kinase (eEF2K), and in myofibroblast differentiation and bleomycin-induced lung fibrosis by targeting targets Smad7 [65,66]. miR-4516 has been shown to down regulate the STAT3-signaling pathway, which results from the induction of UV-induced apoptosis in keratinocytes [67]. miR-6087 is incorporated in human adipose mesenchymal stem cell-derived exosomes, which exhibited an anti-proliferative effect for ovarian cancer cell lines [68]. miR-7704 is upregulated in macrophages treated with a combination of interleukin-27 (IL-27) and macrophage colony stimulating factor. The same study identified putative targets genes for miR-7704, such as GF0D1, the membrane-associated RING-CH3 (MARCH3), and the Src homology 2 domain containing transforming protein C3 (SHC3). miR-7704 has been shown to target the open reading frames of a number of viruses, including Herpes simplex virus (HSV)-1, HSV-2, and human herpesvirus (HHV)-8, suggesting that miR-7704 may partially contribute to the anti-viral properties of IL-27 against these viruses [69]. Although the functional properties of these miRNAs in EBV’s lifecycle remain unclear, EBV may exploit the mechanism of active sorting of these miRNAs, which possess tumor suppressive, anti-viral, or anti-apoptotic functions, into exosomes to induce EBV-associated tumors. Moreover, these unique miRNAs could also lead to potential biomarkers, enabling earlier and more accurate diagnoses and accelerating the development of therapeutics for EBV-associated tumors. It is also essential to perform NGS for other sets of BL cell lines to confirm the generality of our findings.

Previously we demonstrated that exosomes derived from all three Mutu cells were internalized into the target cells via caveolae-dependent endocytosis in a similar fashion. Moreover, we observed that exosomes (III) up-regulated proliferation and expression of intercellular adhesion molecule 1 (ICAM-1) in the recipient cells more significantly than exosome (−) and exosome (I). We also identified LMP1 as a gene responsible for induction of ICAM-1 expression [56]. 

Taken together, our findings indicate that EBV infection, in particular in type III latency, modulates the biogenesis of exosomes and expression profile of exosomal miRNAs, which along with LMP1 may contribute to the induction of EBV-associated tumors by modulating cell and virus functions.

## 4. Materials and Methods

### 4.1. Cell Culture 

Mutu I and Mutu III cells, which are type I and type III latency EBV-infected B cell lines, respectively, were established from the identical BL tumor [70]. EBV-negative subclone (Mutu^−^) was isolated from Mutu I by the limiting dilution methods [71]. BJAB cell is an EBV-negative BL cell line [72]. Namalwa cell is a BL cell line possessing two copies of EBV DNA integrated into host genome [73,74]. Mutu^−^, Mutu I, Mutu III, BJAB, and Namalwa cells were maintained in RPMI-1640 medium containing 10% fetal bovine serum (FBS) and antibiotics. EBV-negative human GC epithelial AGS [75,76] were grown in high-glucose Dulbecco’s modified Eagle’s medium (DMEM) containing 10% FBS and antibiotics. 

### 4.2. Purification of Exosomes

For the purification of exosomes, Mutu^−^, Mutu I, and Mutu III cells (2 × 10^5^/mL) were grown in 200 mL RPMI 1640 medium containing 10% exosome-depleted FBS, which was prepared by centrifugation at 25,000 rpm for 4 h at 4 °C, until they are confluent. Culture medium containing exosomes was harvested and centrifuged at 1500 rpm for 10 min and at 6000 rpm for 20 min at room temperature to remove cells and cell debris, respectively. The exosomes were pelleted by centrifugation at 25,000 rpm for 1 h at 4 °C with an SW28 rotor (Beckman, Fullerton, CA, USA). The pelleted exosomes were resuspended in 50 µL TNE buffer [10 mM Tris-HCl (pH 7.6), 100 mM NaCl, 1 mM EDTA] overnight. A 5 μL aliquot of the fraction containing exosomes were confirmed by western blot with mouse anti-CD63 monoclonal antibody (clone MEM-250, Abnova, Taipei, Taiwan, 1:1000 dilution), mouse anti-LMP1 monoclonal antibody (clone S12, kindly provided by Dr. Teruhito Yasui, National Institutes of Biomedical Innovation, Health and Nutrition, 1:10,000 dilution), mouse anti-Alix monoclonal antibody (clone 3A9, BioLegend, San Diego, CA, USA, 1:1000 dilution), rabbit anti-calnexin polyclonal antibody (Cell Signaling Technology, Trask Lane, MA, USA, 1:1,000 dilution), and rabbit anti-GM130 monoclonal antibody (clone D6B1, Cell Signaling Technology, 1:1000 dilution). The total protein concentration in the fractions containing exosomes was determined by the Bradford protein assay (Bio-Rad Laboratories, Hercules, CA, USA). 

### 4.3. Electron Microscopy

A 6-μL aliquot of the fractions containing exosomes was absorbed onto glow-discharged 300-mesh heavy-duty carboncoated Cu grids (Veco grids, Nisshin EM, Tokyo, Japan) for 2 min and the excess was blotted on filter paper (Whatman, GE Healthcare, Piscataway, NJ, USA). The grids were then washed twice with MilliQ water and negatively stained with 2% uranyl acetate. Data were collected using an HT7700 transmission electron microscope (Hitachi, Tokyo, Japan) operating at 80 kV and 10,000× magnification.

### 4.4. Quantitative PCR

A 10 μL aliquot of exosomes was treated with 5 U DNase at 37 °C for 15 min. DNase was inactivated by incubation at 70 °C for 5 min in the presence of 2.5 mM EDTA. DNA was extracted by use of the DNeasy Blood & Tissue Kit (Qiagen Hilden, Germany). As positive control, progeny EBV was obtained by treatment of EBV-infected Akata cells (Akata^+^) with 1% goat anti-human IgG (αhIgG) (DAKO, Glostrup, Denmark) for 48 h [51,77,78]. After treatment with DNase I, EBV DNA in the supernatant was isolated as described above. For detection of EBV DNA, real-time PCR was carried out as previously described [29,79] with slight modifications by means of sense (CGGAAGCCCTCTGGACTTC) and antisense (CCCTGTTTATCCGATGGAATG) oligonucleotides and probe (TGTACACGCACGAGAAATGCGCC) specific for the EBV-encoded BARF5 sequence. As an internal control, cellular Rhodopsin was analyzed using sense (ATCAGGAACCATTGCCACGTCCTA) and antisense (AGGCCAAAGATGGACACACAGAGT) oligonucleotides and probe (AGCCTCTAGTTTCCAGAAGCTGCACA). The copy number of EBV genomes were quantified with a standard curve of dilution of the plasmid encoding EBV BARF5 gene followed by the normalization by the number of cellular genomes quantified against a standard curve of dilution of the plasmid encoding Rhodopsin.

### 4.5. Fluorescent In Situ Hybridization (FISH)

FISH analysis was performed as described previously [62,80]. Briefly Mutu cells were treated with 0.075 M KCl for 20 min at 37 °C, fixed in methanol:acetic acid (3:1) for 30 min at room temperature and spread on the slides. Slides were treated with 4 × SSC (1 × SSC; 0.15 M NaCl, 0.015 M sodium citrate) containing 0.5% (*v*/*v*) nonidet P-40 for 30 min at 37 °C, dehydrated in a cold ethanol series (70, 80, 90%) for 2 min each, air dried and denatured in 70% formamide-2 × SSC for 2 min at 72 °C. Slides were dehydrated in a cold ethanol series and air dried. Hybridization probes for detection of EBV genomes were generated by nick translation with the plasmid containing EBV BamHI-WWYH fragment using biotin-11-dUTP (Roche, Basel, Switzerland). 20 µg of a probe was precipitated by ethanol in the presence of 6 µg salmon sperm DNA (Eppendorf, Hamburg, Germany) and 4 µg human Cot-1 DNA (Thermo Fisher Scientific, Waltham, MA, USA), resuspended in hybridization buffer (2 × SSC, 50% formamide, 10% dextran sulfate) and incubated for 10 min at 70 °C, for 5 min at 4 °C and for 1 h at 37 °C. A hybridization mix containing 5 ng probe was placed on each sample and incubated overnight at 37 °C in a moist chamber. Slides were washed in 2 × SSC containing 50% formamide for 30 min at 50 °C and in 2 × SSC for 30 min at 50 °C. After blocking in 4 × SSC containing 1% BSA, the hybridized probe was revealed by incubation with streptavidin conjugated to FITC (Sigma-Aldrich, St. Louis, MO, USA) for 30 min at 37 °C. Slides were washed twice in 4 × SSC containing 0.05% Triton X-100 for 5 min at room temperature. The nucleus was counterstained with Hoechst 33342 (Cell Signaling Technology). The copy numbers of EBV genome were analyzed by a confocal laser scanning microscope. (Fluoview FV10i, Olympus, Tokyo, Japan) and acquired by using FV10-ASW software Ver. 4.2 (Olympus, Tokyo, Japan). 

### 4.6. Immunofluorescence Staining

Mutu cells were fixed with 4% paraformaldehyde in PBS for 10 min at room temperature, permeabilized with PBS containing 0.05% Triton X-100 for 10 min at room temperature and blocked in PBS containing 1% bovine serum albumin (BSA) for 20 min at room temperature. The cells were incubated with rabbit anti-CD63 polyclonal antibody (Abcam, Cambridge, UK, 1:200 dilution) and anti-Alix monoclonal antibody (BioLegend, 1:200 dilution) for 1 h at room temperature. After washing twice in PBS, the cells were incubated with Alexa Fluor 488-labeled anti-mouse IgG (Thermo Fisher Scientific, 1:1000 dilution) for 1 h at room temperature. After washing twice in PBS, the nuclei were counterstained with Hoechst 33342. Images were collected by a confocal laser scanning microscope. Same samples were quantitatively analyzed by means of flowcytometry.

### 4.7. RNA Extraction

Total RNA was extracted from cells or exosomes using the mirVana miRNA Isolation Kit (Thermo Fisher Scientific). The extracted RNA was analyzed using small RNA chips on a 2100 Bioanalyzer (Agilent Technologies, Santa Clara, CA, USA). Small RNA profiles contained small RNA consists on between 4 and 40 nucleotides in length, which is consistent with miRNA.

### 4.8. Next-Generation Sequencing (NGS)

Small RNA library for sequencing was prepared by the TruSeq Small RNA-Seq Sample Prep Kit (Illumina, San Diego, CA, USA). The quality and yield after sample preparation were analyzed by use of the Bioanalyzer with a High Sensitivity DNA kit (Agilent Technologies) and corresponded to the expected 150 bp. DNA sequencing was carried out using Miseq (Illumina) with MiSeq reagent kit v3. Sequence reads were analyzed with CLC Genomics Workbench (version 11.0; Qiagen). After adaptor trimming, reads of less than 15 or more than 26 nucleotides in length were removed, and all reads of 15–25 nucleotides in length were analyzed against miRBase release 21 retrieved from the miRNA database (http://www.mirbase.org/). Homo_sapiens.GRCh37.57.ncrna was used as a comprehensive noncoding RNA database (http://www.ncrna.org/). Human tRNA was annotated using GRCh37/hg19 tRNA database (GtRNAdb, http://gtrnadb.ucsc.edu/). Reads matching pre-miRNA were counted as miRNA reads. The ratio of read numbers of mature miRNAs in total annotated miRNAs were analyzed between the samples. NSG analysis was duplicated for intracellular miRNAs derived from Mutu cells, and exosomal miRNAs from Mutu III, and triplicated for exosomal miRNAs derived from Mutu^−^ and Mutu I.

### 4.9. Exosome Transfer Assay

Exosome transfer assay was performed as described previously [56]. Briefly, AGS cells (5 × 10^4^/well) were grown in the basolateral chamber of 24-well trans-well plate (Corning, Toledo, NY, USA). Mutu^−^, Mutu I, or Mutu III (1 × 10^5^, each) were added to the membrane inserts with pore size of 0.4 μm and incubated for 3 days. For the inhibitor treatment of exosome secretion, DMSO or 10 µM GW4869 (Sigma-Aldrich) was added to the coculture and incubated for 3 days Total RNA was isolated from AGS cells after cocultivation and subjected to Stem-loop real-time PCR.

### 4.10. Stem-Loop Real-Time PCR

For analysis of copy numbers of individual EBV miRNAs, total RNA was reversed transcribed using TaqMan MicroRNA Reverse Transcription Kit (Thermo Fisher Scientific) followed by real-time PCR reaction with TaqMan Universal Master Mix, no UNG AmpErase (Thermo Fisher Scientific) as described previously [29,81]. 

### 4.11. Accession Number

Sequence data of the small RNAs analyzed by NGS in this study were deposited in the DNA Data Bank of Japan (DDBJ; accession number: DRA006656, Bioproject PRJDB6853).

## 5. Conclusions 

Here, we characterized the expression profile of exosomal miRNAs derived from EBV-negative, type I and type III latently infected cell lines, which were established from the same African BL tumor. Taken together, our findings indicate that EBV infection in type III latency modulates the biogenesis of exosomes and the profile of exosomal miRNAs, potentially contributing to induction of EBV-associated tumors by phenotypic modifications in adjacent recipient cells. 

## Figures and Tables

**Table 1:** Read counts of cellular and exosomal miRNAs by NGS.

Samples		Raw Reads	Annotated with Database (% Total Annotated Reads)				
			Annotated Reads (% Raw Reads)	miRBase		Homo_Sapiens. GRCh38.ncrna	Human-Trna
				Homo sapiens	EBV		
Mutu^−^	cell	119,769	42,692 (35.65)	11,958 (28.01)	1 (0.00)	28,749 (67.34)	1984 (4.65)
	exosome	66,018	25,690 (38.91)	12,040 (46.87)	1 (0.00)	13,051 (50.80)	598 (2.33)
Mutu I	cell	26,388	10,934 (41.44)	6459 (59.07)	461 (7.14)	3612 (33.03)	402 (3.68)
	exosome	535,527	64,085 (11.97)	32,041 (50.00)	1233 (3.85)	28,267 (44.11)	2544 (3.97)
Mutu III	cell	1,559,462	1,087,053 (69.71)	769,785 (70.81)	157,016 (14.44)	137,381 (12.64)	22,871 (2.10)
	exosome	2,445,816	522,266 (21.35)	80,434 (15.40)	6489 (8.07)	422,846 (80.96)	12,497 (2.39)

**Table 2:** Top 40 miRNAs detected in Mutu cell lines.

	Mutu^−^		Mutu I		Mutu III	
	miRNA	% *^,1^	miRNA	%	miRNA	%
1	miR-92a-1//miR-92a-2-3p	18.20	miR-92a-1//miR-92a-2-3p	34.31	miR-148a-3p	22.06
2	miR-378a-3p	13.65	miR-182-5p	7.90	miR-182-5p	18.70
3	miR-28-3p	6.33	miR-BART10-3p	3.74	miR-92a-1//miR-92a-2-3p	15.03
4	miR-181a-2//miR-181a-1-5p	6.15	miR-378a-3p	3.62	miR-BART10-3p	8.48
5	miR-182-5p	5.98	miR-148a-3p	3.06	miR-181a-2//miR-181a-1-5p	4.27
6	miR-486-1//miR-486-2-5p	4.66	miR-25-3p	3.04	miR-181b-1//miR-181b-2-5p	2.89
7	miR-181b-1//miR-181b-2-5p	4.33	let-7f-1//let-7f-2-5p	2.68	miR-25-3p	2.71
8	miR-148a-3p	3.73	miR-191-5p	2.13	miR-BART6-3p	2.35
9	miR-25-3p	3.00	miR-28-3p	2.04	miR-378a-3p	2.02
10	miR-101-1//miR-101-2-3p	2.74	miR-103a-2//miR-103a-1-3p	1.95	miR-BART17-5p	1.62
11	miR-1275-5p	2.64	miR-21-3p	1.95	miR-BART7-5p	1.40
12	miR-21-3p	2.29	let-7a-1//let-7a-2//let-7a-3-5p	1.91	miR-191-5p	1.34
13	miR-103a-2//miR-103a-1-3p	2.18	miR-30e-3p	1.79	miR-21-5p	1.13
14	miR-30e-3p	2.11	miR-181a-2//miR-181a-1-5p	1.60	miR-30e-3p	1.10
15	miR-191-5p	1.85	miR-128-1-5p	1.51	miR-21-3p	1.04
16	miR-140-3p	1.27	miR-101-1//miR-101-2-3p	1.48	let-7f-1//let-7f-2-5p	0.97
17	miR-423-5p	0.98	miR-486-1//miR-486-2-5p	1.44	miR-BART22-3p	0.92
18	miR-128-1-5p	0.85	miR-181b-1//miR-181b-2-5p	1.41	miR-BART11-3p	0.91
19	miR-423-3p	0.65	miR-106b-3p	1.18	miR-181a-2-3p	0.78
20	miR-210-3p	0.65	miR-BART7-5p	1.07	miR-103a-2//miR-103a-1-3p	0.78
21	miR-769-5p	0.62	miR-320a-3p	1.03	miR-155-5p	0.75
22	let-7i-5p	0.61	let-7i-5p	0.92	miR-101-1//miR-101-2-3p	0.73
23	miR-30d-5p	0.60	miR-1307-3p	0.88	miR-BART13-5p	0.61
24	miR-106b-3p	0.59	miR-140-3p	0.76	miR-148a-5p	0.44
25	miR-186-5p	0.58	miR-423-5p	0.70	miR-30d-5p	0.39
26	miR-142-3p	0.57	miR-30d-5p	0.68	let-7a-1//let-7a-2//let-7a-3-5p	0.37
27	miR-184-3p	0.51	miR-1275-5p	0.67	miR-106b-3p	0.33
28	miR-320a-3p	0.50	miR-877-5p	0.64	let-7c-5p	0.27
29	miR-21-5p	0.46	miR-16-1//miR-16-2-5p	0.58	miR-125b-2-3p	0.27
30	let-7a-1//let-7a-2//let-7a-3-5p	0.44	miR-186-5p	0.56	miR-423-5p	0.26
31	miR-1307-3p	0.42	miR-577-5p	0.56	miR-26a-1//miR-26a-2-5p	0.21
32	miR-143-3p	0.41	miR-142-3p	0.53	miR-7974-3p	0.18
33	miR-181a-1-3p	0.38	miR-BART11-3p	0.51	miR-146b-5p	0.18
34	miR-296-3p	0.37	miR-BART6-3p	0.45	miR-486-1//miR-486-2-5p	0.17
35	miR-1285-1//miR-1285-2-3p	0.32	let-7g-5p	0.42	let-7i-5p	0.17
36	miR-27b-3p	0.31	miR-93-5p	0.39	miR-27b-3p	0.17
37	miR-151a-5p	0.27	miR-423-3p	0.38	miR-16-1//miR-16-2-5p	0.17
38	miR-1260a-5p	0.25	miR-143-3p	0.33	miR-181a-1-3p	0.14
39	miR-877-5p	0.25	miR-151a-5p	0.30	miR-532-5p	0.14
40	miR-130b-3p	0.23	miR-98-5p	0.29	miR-92a-1-5p	0.14

*^,1^, % matured miRNA in total annotated miRNAs.

**Table 3:** Top 40 miRNAs detected in exosomes derived from Mutu cell lines.

	Exosome (−)			Exosome (I)			Exosome (III)		
	miRNA	% *^,1^	-Fold *^,2^	miRNA	%	-Fold	miRNA	%	-Fold
1	miR-92a-1//miR-92a-2-3p	43.77	2.40	miR-92a-1//miR-92a-2-3p	11.8	0.35	miR-92a-1//miR-92a-2-3p	35.16	2.34
2	miR-10b-5p	8.16	-	miR-486-1//miR-486-2-5p	9.39	6.53	miR-486-1//miR-486-2-5p	19.55	112.19
3	miR-378a-3p	7.51	0.55	miR-181a-2//miR-181a-1-5p	7.29	4.54	miR-182-5p	5.00	0.27
4	miR-486-1//miR-486-2-5p	6.60	1.42	miR-378a-3p	6.65	1.84	miR-10b-5p	4.40	699.66
5	miR-25-3p	5.67	1.89	miR-186-5p	5.03	8.98	miR-BART10-3p	4.20	0.50
6	miR-181b-1//miR-181b-2-5p	3.77	0.87	miR-16-1//miR-16-2-5p	4.82	8.37	miR-181a-2//miR-181a-1-5p	3.37	0.79
7	miR-28-3p	2.90	0.46	miR-191-5p	4.35	2.04	miR-148a-3p	2.76	0.13
8	miR-182-5p	2.68	0.45	miR-27b-3p	2.81	11.59	miR-7704-5p	2.34	758.35
9	miR-181a-2//miR-181a-1-5p	1.80	0.29	miR-28-3p	2.70	1.32	miR-378a-3p	2.30	1.14
10	miR-143-3p	1.33	3.27	miR-30e-5p	2.61	11.51	miR-25-3p	2.28	0.84
11	miR-191-5p	1.07	0.58	miR-142-5p	2.50	20.64	miR-181b-1//miR-181b-2-5p	2.18	0.75
12	miR-148a-3p	0.91	0.24	miR-143-3p	2.32	6.98	miR-191-5p	1.90	1.42
13	miR-320a-3p	0.79	1.59	miR-26a-1//miR-26a-2-5p	2.05	22.54	miR-423-5p	1.59	6.02
14	miR-423-5p	0.70	0.71	miR-21-5p	2.04	9.62	miR-BART17-5p	1.45	0.90
15	miR-21-3p	0.63	0.28	miR-30d-5p	1.99	2.92	miR-BART11-3p	1.39	1.53
16	miR-30e-3p	0.63	0.30	miR-10b-5p	1.93	25.44	miR-BART13-5p	0.79	1.29
17	let-7a-1//let-7a-2//let-7a-3-5p	0.61	1.39	miR-25-3p	1.84	0.61	miR-BART6-3p	0.73	0.31
18	miR-106b-3p	0.57	0.97	miR-22-3p	1.63	7.18	miR-30d-5p	0.62	1.58
19	miR-877-5p	0.57	2.24	miR-93-5p	1.36	3.45	miR-181a-2-3p	0.40	0.50
20	miR-103a-2//miR-103a-1-3p	0.51	0.24	let-7a-1//let-7a-2//let-7a-3-5p	1.33	0.70	let-7f-1//let-7f-2-5p	0.38	0.39
21	miR-577-5p	0.49	3.84	miR-148a-3p	1.15	0.38	miR-103a-2//miR-103a-1-3p	0.36	0.46
22	miR-101-1//miR-101-2-3p	0.48	0.17	miR-15a-5p	1.07	17.69	miR-BART7-5p	0.36	0.26
23	let-7f-1//let-7f-2-5p	0.44	3.48	miR-BART11-3p	0.98	1.90	miR-1307-3p	0.36	4.03
24	miR-423-3p	0.41	0.63	miR-BART7-5p	0.93	0.87	miR-155-5p	0.32	0.43
25	miR-140-3p	0.39	0.31	let-7f-1//let-7f-2-5p	0.89	0.33	miR-4516-5p	0.31	406.30
26	miR-432-5p	0.35	0.57	miR-127-3p	0.84	55.84	miR-106b-3p	0.26	0.79
27	let-7i-5p	0.33	1.46	miR-151a-3p	0.76	8.32	miR-BHRF1-1-5p	0.26	8.01
28	miR-130b-3p	0.30	0.73	miR-BART10-3p	0.68	0.18	miR-877-5p	0.24	22.33
29	miR-1307-3p	0.27	0.46	miR-182-5p	0.68	0.09	miR-320a-3p	0.23	8.79
30	miR-186-5p	0.27	1.73	miR-126-5p	0.63	10.49	let-7a-1//let-7a-2//let-7a-3-5p	0.20	0.55
31	miR-93-5p	0.25	7.05	miR-1246-5p	0.63	41.65	miR-21-3p	0.19	0.18
32	miR-4516-5p	0.21	0.35	miR-101-1//miR-101-2-3p	0.58	0.39	miR-6087-5p	0.19	-
33	miR-30d-5p	0.19	0.97	miR-151a-5p	0.57	1.89	miR-BART22-3p	0.18	0.19
34	miR-1304-3p	0.17	0.27	miR-103a-2//miR-103a-1-3p	0.57	0.29	miR-423-3p	0.17	2.11
35	miR-1246-5p	0.17	19.26	miR-192-5p	0.54	8.97	miR-30e-3p	0.17	0.15
36	miR-210-3p	0.17	0.26	miR-128-1//miR-128-2-3p	0.47	30.86	miR-125b-2-3p	0.16	0.58
37	miR-16-1//miR-16-2-5p	0.16	1.07	miR-425-5p	0.44	29.35	let-7c-5p	0.15	0.55
38	miR-320b-1//miR-320b-2-3p	0.15	8.54	let-7g-5p	0.44	1.04	miR-21-5p	0.14	0.12
39	miR-660-5p	0.15	4.27	miR-1307-5p	0.43	14.05	miR-BART19-5p	0.14	1.99
40	miR-1285-1//miR-1285-2-3p	0.15	0.49	miR-181b-1//miR-181b-2-5p	0.42	0.30	miR-143-3p	0.13	25.43

* ^1^, % matured miRNA in total annotated miRNAs; * ^2^, ratio of exosomal miRNA to cellular miRNA.

**Table 4:** EXOmotifs in highly concentrated miRNA into exosomes.

miRNA		% Mature Annotated		Concentrate (-Fold)	Sequence	EXOmotifs
		Cell	Exosome			
Cell	miR-6087-5p	N/D	13.92	-	**TGAG**GCGGGGG**G****GCG**AGC	8
	miR-7704-5p	0.003	2.34	758.35	CGGGGTC**GGCG**GCGACGTG	3
	miR-10b-5p	0.006	4.40	699.66	TACCCTGTAGAACCGAATTTGTG	1
	miR-4516-5p	0.001	0.31	406.30	GGGAGAAGGGTC**GGGG**C	3
	miR-486-1//miR-486-2-5p	0.174	19.55	112.19	TCCTGTACTGAGCTGC**C****CCG**AG	4
EBV	miR-BART7-3p	0.015	0.13	8.53	CATCATAGTCCAGTGTCCAGGG	2
	miR-BHRF1-1-5p	0.033	0.26	8.01	TAACCTGATCAGC**CCC****G**GAGTT	2
	miR-BART2-5p	0.015	0.09	6.27	TATTTTCTGCATTCGCCCTTGC	1

EXOmotifs are shown with under lines, double under lines, or in bold letters.

## Supplementary Materials

The following are available online at http://www.mdpi.com/2072-6694/10/7/237/s1, Table S1: Summary of read counts of miRNAs, Table S2: Summary of expression profile of miRNAs, Table S3: Summary of expression profile of EBV-encoded miRNAs.

## Abbreviations

EBV	Epstein–Barr virus
BL	Burkitt lymphoma
NGS	Next-generation sequence
miRNA	microRNA
MVB	multivesicular body

## References

[1] Longnecker R.M., Kieff E., Cohen J.I., Knipe M., Howley P.M. (2013). Epstein–Barr virus. Fields Virology.

[2] Raab-Traub N., Dittmer D.P. (2017). Viral effects on the content and function of extracellular vesicles. Nat. Rev. Microbiol..

[3] Meckes D.G. (2015). Exosomal communication goes viral. J. Virol..

[4] Wollert T., Hurley J.H. (2010). Molecular mechanism of multivesicular body biogenesis by escrt complexes. Nature.

[5] Johnstone R.M., Adam M., Hammond J.R., Orr L., Turbide C. (1987). Vesicle formation during reticulocyte maturation. Association of plasma membrane activities with released vesicles (exosomes). J. Biol. Chem..

[6] Van Niel G., D’Angelo G., Raposo G. (2018). Shedding light on the cell biology of extracellular vesicles. Nat. Rev. Mol. Cell Biol..

[7] Raposo G., Stoorvogel W. (2013). Extracellular vesicles: Exosomes, microvesicles, and friends. J. Cell Biol..

[8] Lasser C., Alikhani V.S., Ekstrom K., Eldh M., Paredes P.T., Bossios A., Sjostrand M., Gabrielsson S., Lotvall J., Valadi H. (2011). Human saliva, plasma and breast milk exosomes contain RNA: Uptake by macrophages. J. Transl. Med..

[9] Tkach M., Thery C. (2016). Communication by extracellular vesicles: Where we are and where we need to go. Cell.

[10] Vojtech L., Woo S., Hughes S., Levy C., Ballweber L., Sauteraud R.P., Strobl J., Westerberg K., Gottardo R., Tewari M. (2014). Exosomes in human semen carry a distinctive repertoire of small non-coding RNAs with potential regulatory functions. Nucleic Acids Res..

[11] Valadi H., Ekstrom K., Bossios A., Sjostrand M., Lee J.J., Lotvall J.O. (2007). Exosome-mediated transfer of mRNAs and microRNAs is a novel mechanism of genetic exchange between cells. Nat. Cell Biol..

[12] Manterola L., Guruceaga E., Gallego Perez-Larraya J., Gonzalez-Huarriz M., Jauregui P., Tejada S., Diez-Valle R., Segura V., Sampron N., Barrena C. (2014). A small noncoding RNA signature found in exosomes of gbm patient serum as a diagnostic tool. Neuro Oncol..

[13] Li M., Zeringer E., Barta T., Schageman J., Cheng A., Vlassov A.V. (2014). Analysis of the RNA content of the exosomes derived from blood serum and urine and its potential as biomarkers. Philos. Trans. R. Soc. Lond. B Biol. Sci..

[14] Batagov A.O., Kurochkin I.V. (2013). Exosomes secreted by human cells transport largely mRNA fragments that are enriched in the 3’-untranslated regions. Biol. Direct..

[15] Bartel D.P. (2018). Metazoan microRNAs. Cell.

[16] Bartel D.P. (2004). MicroRNAs: Genomics, biogenesis, mechanism, and function. Cell.

[17] Kuzembayeva M., Hayes M., Sugden B. (2014). Multiple functions are mediated by the miRNAs of Epstein–Barr virus. Curr. Opin. Virol..

[18] Vereide D.T., Seto E., Chiu Y.F., Hayes M., Tagawa T., Grundhoff A., Hammerschmidt W., Sugden B. (2014). Epstein–Barr virus maintains lymphomas via its miRNAs. Oncogene.

[19] Tagawa T., Albanese M., Bouvet M., Moosmann A., Mautner J., Heissmeyer V., Zielinski C., Lutter D., Hoser J., Hastreiter M. (2016). Epstein–Barr viral miRNAs inhibit antiviral cd4+ t cell responses targeting il-12 and peptide processing. J. Exp. Med..

[20] Seto E., Moosmann A., Gromminger S., Walz N., Grundhoff A., Hammerschmidt W. (2010). Micro RNAs of Epstein–Barr virus promote cell cycle progression and prevent apoptosis of primary human b cells. PLoS Pathog..

[21] Feederle R., Linnstaedt S.D., Bannert H., Lips H., Bencun M., Cullen B.R., Delecluse H.J. (2011). A viral microRNA cluster strongly potentiates the transforming properties of a human herpesvirus. PLoS Pathog..

[22] Bernhardt K., Haar J., Tsai M.H., Poirey R., Feederle R., Delecluse H.J. (2016). A viral microRNA cluster regulates the expression of pten, p27 and of a bcl-2 homolog. PLoS Pathog..

[23] Albanese M., Tagawa T., Bouvet M., Maliqi L., Lutter D., Hoser J., Hastreiter M., Hayes M., Sugden B., Martin L. (2016). Epstein–Barr virus microRNAs reduce immune surveillance by virus-specific cd8+ t cells. Proc. Natl. Acad. Sci. USA.

[24] Shinozaki-Ushiku A., Kunita A., Isogai M., Hibiya T., Ushiku T., Takada K., Fukayama M. (2015). Profiling of virus-encoded microRNAs in Epstein–Barr virus-associated gastric carcinoma and their roles in gastric carcinogenesis. J. Virol..

[25] Marquitz A.R., Mathur A., Chugh P.E., Dittmer D.P., Raab-Traub N. (2014). Expression profile of microRNAs in Epstein–Barr virus-infected ags gastric carcinoma cells. J. Virol..

[26] Kang D., Skalsky R.L., Cullen B.R. (2015). Ebv bart microRNAs target multiple pro-apoptotic cellular genes to promote epithelial cell survival. PLoS Pathog..

[27] Kanda T., Miyata M., Kano M., Kondo S., Yoshizaki T., Iizasa H. (2015). Clustered microRNAs of the Epstein–Barr virus cooperatively downregulate an epithelial cell-specific metastasis suppressor. J. Virol..

[28] Cai L.M., Lyu X.M., Luo W.R., Cui X.F., Ye Y.F., Yuan C.C., Peng Q.X., Wu D.H., Liu T.F., Wang E. (2015). Ebv-mir-bart7-3p promotes the emt and metastasis of nasopharyngeal carcinoma cells by suppressing the tumor suppressor pten. Oncogene.

[29] Pratt Z.L., Kuzembayeva M., Sengupta S., Sugden B. (2009). The microRNAs of Epstein–Barr virus are expressed at dramatically differing levels among cell lines. Virology.

[30] Gallo A., Vella S., Miele M., Timoneri F., Di Bella M., Bosi S., Sciveres M., Conaldi P.G. (2017). Global profiling of viral and cellular non-coding RNAs in Epstein–Barr virus-induced lymphoblastoid cell lines and released exosome cargos. Cancer Lett..

[31] Cameron J.E., Fewell C., Yin Q., McBride J., Wang X., Lin Z., Flemington E.K. (2008). Epstein–Barr virus growth/latency iii program alters cellular microRNA expression. Virology.

[32] Frixa T., Donzelli S., Blandino G. (2015). Oncogenic microRNAs: Key players in malignant transformation. Cancers.

[33] Pegtel D.M., van de Garde M.D., Middeldorp J.M. (2011). Viral miRNAs exploiting the endosomal-exosomal pathway for intercellular cross-talk and immune evasion. Biochim. Biophys. Acta.

[34] Pegtel D.M., Cosmopoulos K., Thorley-Lawson D.A., van Eijndhoven M.A., Hopmans E.S., Lindenberg J.L., de Gruijl T.D., Wurdinger T., Middeldorp J.M. (2010). Functional delivery of viral miRNAs via exosomes. Proc. Natl. Acad. Sci. USA.

[35] Lin Z., Swan K., Zhang X., Cao S., Brett Z., Drury S., Strong M.J., Fewell C., Puetter A., Wang X. (2016). Secreted oral epithelial cell membrane vesicles induce Epstein–Barr virus reactivation in latently infected b cells. J. Virol..

[36] Janas T., Janas M.M., Sapon K., Janas T. (2015). Mechanisms of RNA loading into exosomes. FEBS Lett..

[37] Zhang J., Li S., Li L., Li M., Guo C., Yao J., Mi S. (2015). Exosome and exosomal microRNA: Trafficking, sorting, and function. Genom. Proteom. Bioinform..

[38] Wong A.M., Kong K.L., Tsang J.W., Kwong D.L., Guan X.Y. (2012). Profiling of Epstein–Barr virus-encoded microRNAs in nasopharyngeal carcinoma reveals potential biomarkers and oncomirs. Cancer.

[39] Qiu J., Cosmopoulos K., Pegtel M., Hopmans E., Murray P., Middeldorp J., Shapiro M., Thorley-Lawson D.A. (2011). A novel persistence associated ebv miRNA expression profile is disrupted in neoplasia. PLoS Pathog..

[40] Piccaluga P.P., Navari M., De Falco G., Ambrosio M.R., Lazzi S., Fuligni F., Bellan C., Rossi M., Sapienza M.R., Laginestra M.A. (2016). Virus-encoded microRNA contributes to the molecular profile of ebv-positive burkitt lymphomas. Oncotarget.

[41] Navari M., Etebari M., De Falco G., Ambrosio M.R., Gibellini D., Leoncini L., Piccaluga P.P. (2015). The presence of Epstein–Barr virus significantly impacts the transcriptional profile in immunodeficiency-associated burkitt lymphoma. Front. Microbiol..

[42] Cosmopoulos K., Pegtel M., Hawkins J., Moffett H., Novina C., Middeldorp J., Thorley-Lawson D.A. (2009). Comprehensive profiling of Epstein–Barr virus microRNAs in nasopharyngeal carcinoma. J. Virol..

[43] Andrade T.A., Evangelista A.F., Campos A.H., Poles W.A., Borges N.M., Camillo C.M., Soares F.A., Vassallo J., Paes R.P., Zerbini M.C. (2014). A microRNA signature profile in ebv+ diffuse large b-cell lymphoma of the elderly. Oncotarget.

[44] Amoroso R., Fitzsimmons L., Thomas W.A., Kelly G.L., Rowe M., Bell A.I. (2011). Quantitative studies of Epstein–Barr virus-encoded microRNAs provide novel insights into their regulation. J. Virol..

[45] Sakamoto K., Sekizuka T., Uehara T., Hishima T., Mine S., Fukumoto H., Sato Y., Hasegawa H., Kuroda M., Katano H. (2017). Next-generation sequencing of miRNAs in clinical samples of Epstein–Barr virus-associated b-cell lymphomas. Cancer Med..

[46] Motsch N., Alles J., Imig J., Zhu J., Barth S., Reineke T., Tinguely M., Cogliatti S., Dueck A., Meister G. (2012). MicroRNA profiling of Epstein–Barr virus-associated NK/T-cell lymphomas by deep sequencing. PLoS ONE.

[47] Imig J., Motsch N., Zhu J.Y., Barth S., Okoniewski M., Reineke T., Tinguely M., Faggioni A., Trivedi P., Meister G. (2011). MicroRNA profiling in Epstein–Barr virus-associated b-cell lymphoma. Nucleic Acids Res..

[48] Chen S.J., Chen G.H., Chen Y.H., Liu C.Y., Chang K.P., Chang Y.S., Chen H.C. (2010). Characterization of Epstein–Barr virus miRNAome in nasopharyngeal carcinoma by deep sequencing. PLoS ONE.

[49] Alles J., Menegatti J., Motsch N., Hart M., Eichner N., Reinhardt R., Meister G., Grasser F.A. (2016). MiRNA expression profiling of Epstein–Barr virus-associated nktl cell lines by illumina deep sequencing. FEBS Open Biol..

[50] Hoshina S., Sekizuka T., Kataoka M., Hasegawa H., Hamada H., Kuroda M., Katano H. (2016). Profile of exosomal and intracellular microRNA in gamma-herpesvirus-infected lymphoma cell lines. PLoS ONE.

[51] Takada K., Horinouchi K., Ono Y., Aya T., Osato T., Takahashi M., Hayasaka S. (1991). An Epstein–Barr virus-producer line akata: Establishment of the cell line and analysis of viral DNA. Virus Genes.

[52] Escola J.M., Kleijmeer M.J., Stoorvogel W., Griffith J.M., Yoshie O., Geuze H.J. (1998). Selective enrichment of tetraspan proteins on the internal vesicles of multivesicular endosomes and on exosomes secreted by human b-lymphocytes. J. Biol. Chem..

[53] Baietti M.F., Zhang Z., Mortier E., Melchior A., Degeest G., Geeraerts A., Ivarsson Y., Depoortere F., Coomans C., Vermeiren E. (2012). Syndecan-syntenin-alix regulates the biogenesis of exosomes. Nat. Cell Biol..

[54] Lotvall J., Hill A.F., Hochberg F., Buzas E.I., Di Vizio D., Gardiner C., Gho Y.S., Kurochkin I.V., Mathivanan S., Quesenberry P. (2014). Minimal experimental requirements for definition of extracellular vesicles and their functions: A position statement from the international society for extracellular vesicles. J. Extracell. Vesicles.

[55] Verweij F.J., van Eijndhoven M.A., Hopmans E.S., Vendrig T., Wurdinger T., Cahir-McFarland E., Kieff E., Geerts D., van der Kant R., Neefjes J. (2011). Lmp1 association with cd63 in endosomes and secretion via exosomes limits constitutive nf-kappab activation. EMBO J..

[56] Nanbo A., Kawanishi E., Yoshida R., Yoshiyama H. (2013). Exosomes derived from Epstein–Barr virus-infected cells are internalized via caveola-dependent endocytosis and promote phenotypic modulation in target cells. J. Virol..

[57] Meckes D.G., Shair K.H., Marquitz A.R., Kung C.P., Edwards R.H., Raab-Traub N. (2010). Human tumor virus utilizes exosomes for intercellular communication. Proc. Natl. Acad. Sci. USA.

[58] Hurwitz S.N., Nkosi D., Conlon M.M., York S.B., Liu X., Tremblay D.C., Meckes D.G. (2017). Cd63 regulates Epstein–Barr virus lmp1 exosomal packaging, enhancement of vesicle production, and noncanonical nf-kappab signaling. J. Virol..

[59] Villarroya-Beltri C., Gutierrez-Vazquez C., Sanchez-Madrid F., Mittelbrunn M. (2013). Analysis of microRNA and protein transfer by exosomes during an immune synapse. Methods Mol. Biol..

[60] Trajkovic K., Hsu C., Chiantia S., Rajendran L., Wenzel D., Wieland F., Schwille P., Brugger B., Simons M. (2008). Ceramide triggers budding of exosome vesicles into multivesicular endosomes. Science.

[61] Hurwitz S.N., Cheerathodi M.R., Nkosi D., York S.B., Meckes D.G. (2018). Tetraspanin cd63 bridges autophagic and endosomal processes to regulate exosomal secretion and intracellular signaling of Epstein–Barr virus lmp1. J. Virol..

[62] Nanbo A., Sugden A., Sugden B. (2007). The coupling of synthesis and partitioning of ebv’s plasmid replicon is revealed in live cells. EMBO J..

[63] Kosaka N., Iguchi H., Hagiwara K., Yoshioka Y., Takeshita F., Ochiya T. (2013). Neutral sphingomyelinase 2 (nsmase2)-dependent exosomal transfer of angiogenic microRNAs regulate cancer cell metastasis. J. Biol. Chem..

[64] Pekow J., Meckel K., Dougherty U., Butun F., Mustafi R., Lim J., Crofton C., Chen X., Joseph L., Bissonnette M. (2015). Tumor suppressors mir-143 and mir-145 and predicted target proteins api5, erk5, k-ras, and irs-1 are differentially expressed in proximal and distal colon. Am. J. Physiol. Gastrointest. Liver Physiol..

[65] Wang C., Gu S., Cao H., Li Z., Xiang Z., Hu K., Han X. (2016). Mir-877-3p targets smad7 and is associated with myofibroblast differentiation and bleomycin-induced lung fibrosis. Sci. Rep..

[66] Shi Q., Xu X., Liu Q., Luo F., Shi J., He X. (2016). MicroRNA-877 acts as a tumor suppressor by directly targeting eef2k in renal cell carcinoma. Oncol. Lett..

[67] Chowdhari S., Saini N. (2014). Hsa-mir-4516 mediated downregulation of stat3/cdk6/ube2n plays a role in puva induced apoptosis in keratinocytes. J. Cell. Physiol..

[68] Reza A.M., Choi Y.J., Yasuda H., Kim J.H. (2016). Human adipose mesenchymal stem cell-derived exosomal-miRNAs are critical factors for inducing anti-proliferation signalling to a2780 and skov-3 ovarian cancer cells. Sci. Rep..

[69] Swaminathan S., Hu X., Zheng X., Kriga Y., Shetty J., Zhao Y., Stephens R., Tran B., Baseler M.W., Yang J. (2013). Interleukin-27 treated human macrophages induce the expression of novel microRNAs which may mediate anti-viral properties. Biochem. Biophys. Res. Commun..

[70] Gregory C.D., Rowe M., Rickinson A.B. (1990). Different Epstein–Barr virus-b cell interactions in phenotypically distinct clones of a burkitt’s lymphoma cell line. J. Gen. Virol..

[71] Kitagawa N., Goto M., Kurozumi K., Maruo S., Fukayama M., Naoe T., Yasukawa M., Hino K., Suzuki T., Todo S. (2000). Epstein–Barr virus-encoded poly(a)(-) RNA supports burkitt’s lymphoma growth through interleukin-10 induction. EMBO J..

[72] Menezes J., Leibold W., Klein G., Clements G. (1975). Establishment and characterization of an Epstein–Barr virus (EBC)-negative lymphoblastoid b cell line (BJA-b) from an exceptional, ebv-genome-negative african burkitt’s lymphoma. Biomedicine.

[73] Matsuo T., Heller M., Petti L., O’Shiro E., Kieff E. (1984). Persistence of the entire Epstein–Barr virus genome integrated into human lymphocyte DNA. Science.

[74] Lawrence J.B., Villnave C.A., Singer R.H. (1988). Sensitive, high-resolution chromatin and chromosome mapping in situ: Presence and orientation of two closely integrated copies of ebv in a lymphoma line. Cell.

[75] Yoshiyama H., Imai S., Shimizu N., Takada K. (1997). Epstein–Barr virus infection of human gastric carcinoma cells: Implication of the existence of a new virus receptor different from cd21. J. Virol..

[76] Barranco S.C., Townsend C.M., Casartelli C., Macik B.G., Burger N.L., Boerwinkle W.R., Gourley W.K. (1983). Establishment and characterization of an in vitro model system for human adenocarcinoma of the stomach. Cancer Res..

[77] Takada K., Ono Y. (1989). Synchronous and sequential activation of latently infected Epstein–Barr virus genomes. J. Virol..

[78] Takada K. (1984). Cross-linking of cell surface immunoglobulins induces Epstein–Barr virus in burkitt lymphoma lines. Int. J. Cancer.

[79] Vereide D.T., Sugden B. (2011). Lymphomas differ in their dependence on Epstein–Barr virus. Blood.

[80] Nanbo A., Terada H., Kachi K., Takada K., Matsuda T. (2012). Roles of cell signaling pathways in cell-to-cell contact-mediated Epstein–Barr virus transmission. J. Virol..

[81] Yang Y.C., Liem A., Lambert P.F., Sugden B. (2017). Dissecting the regulation of ebv’s bart miRNAs in carcinomas. Virology.

